# A Network Toxicology Framework for Identification of Immune System Disruption by Per- and Polyfluoroalkyl Substance (PFAS) Mixture: In Silico Analysis

**DOI:** 10.3390/jox16030115

**Published:** 2026-06-19

**Authors:** Katarina Baralić, Katarina Vidić, Đurđica Marić, Jovana Živanović, Aleksandra Buha Djordjevic, Marijana Ćurčić, Zorica Bulat, Biljana Antonijević, Danijela Đukić-Ćosić

**Affiliations:** Department of Toxicology “Akademik Danilo Soldatović”, Faculty of Pharmacy, University of Belgrade, Vojvode Stepe 450, 11221 Belgrade, Serbia; vidickatarina55@gmail.com (K.V.); djurdjica.maric@pharmacy.bg.ac.rs (Đ.M.); jovana.zivanovic@pharmacy.bg.ac.rs (J.Ž.); aleksandra.buha@pharmacy.bg.ac.rs (A.B.D.); marijana.curcic@pharmacy.bg.ac.rs (M.Ć.); zorica.bulat@pharmacy.bg.ac.rs (Z.B.); biljana.antonijevic@pharmacy.bg.ac.rs (B.A.); danijela.djukic.cosic@pharmacy.bg.ac.rs (D.Đ.-Ć.)

**Keywords:** PFAS, bioinformatics, toxicity, genes, interactions, phenotype, immunotoxicity

## Abstract

Per- and polyfluoroalkyl substances (PFAS) are persistent, chemically stable compounds widely used in daily life. Perfluorooctanoic acid (PFOA), perfluorononanoic acid (PFNA), perfluorohexanesulfonic acid (PFHxS), and perfluorooctanesulfonic acid (PFOS) were identified as the most relevant PFAS due to their prevalence and toxicity. This study aimed to investigate the immunotoxic mechanisms of a mixture of these PFAS using an in silico approach. Comparative Toxicogenomic Database (CTD), GeneMANIA, CytoHubba (Cytoscape), ToppGene Suite, and Metascape were used for the analysis. A total of 65 immune-related genes were identified as common to all four PFAS, with *IFNG*, *TNF*, *IL1B*, *IL6*, *TYK2*, *CD3E*, *CASP8*, *VAV1*, *ARHGAP4*, and *CARD11* emerging as key hub genes. CTD phenotype analysis indicated immune dysregulation, with decreased humoral and adaptive immune responses in humans and tissue-specific modulation of B- and T-cell activity in mice, while no immune-related phenotypes were observed for PFNA. Network analysis identified functional modules associated with apoptotic and immune signaling, endothelial cell migration and angiogenesis, and shared inflammatory and viral response pathways. Disease enrichment analysis associated PFAS with autoimmune disorders (rheumatoid arthritis, asthma), metabolic conditions, and cardiovascular diseases (experimental diabetes, hypertensive disease). These results highlight PFAS involvement in immune modulation, cytokine signaling, and disease susceptibility.

## 1. Introduction

The immune system plays an essential role in maintaining human health, protecting the body from harmful environmental influences such as microorganisms, radiation, and toxic chemicals [1,2,3]. Under these influences, the immune system may undergo alterations, resulting in a range of outcomes, from allergic and autoimmune reactions caused by hypersensitivity to conditions of immunodeficiency. Although genetic predisposition plays a crucial role in the pathogenesis of these diseases, the impact of environmental factors is undeniable.

Key external modulators of immune system function include chemicals such as organic solvents [4], heavy metals [5], pesticides, polycyclic aromatic hydrocarbons [6], polychlorinated biphenyls [7,8], and per- and polyfluoroalkyl substances (PFAS) [8].

PFAS represent a heterogeneous group of synthetic compounds defined by the Organization for Economic Cooperation and Development (OECD) as substances containing at least one fully fluorinated methyl or methylene carbon atom without hydrogen, chlorine, bromine, or iodine [9,10]. All PFAS are either intrinsically extremely persistent or degrade in the environment into compounds with similarly high persistence. Their chemical stability [11], ability to travel long distances [12], accumulation in living organisms, and increased concentration along the food chain are the main reasons why this group of compounds is increasingly referred to as “forever chemicals” [13]. Owing to their amphiphilic nature (combining both hydrophilic and hydrophobic properties) and their remarkable resistance to heat, water, and oil, PFAS have been extensively incorporated into a wide variety of industrial applications and consumer products for decades [14]. In addition to the low-molecular-weight PFAS investigated in the present study, the broader PFAS family also includes polymeric compounds, such as fluoropolymers and fluorinated surfactants, which are used in coating materials, non-stick products, and fire-fighting foams. These polymers may contain leachable non-polymeric PFAS, which can be released into the environment and subsequently contribute to human exposure [15,16].

**Figure 1:**
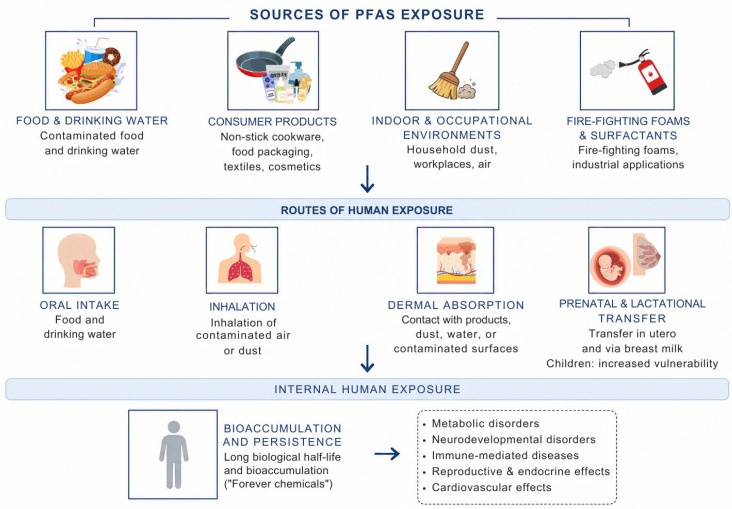
Schematic overview of the major sources, exposure routes, and potential health effects associated with human exposure to per- and polyfluoroalkyl substances (PFAS).

Representative applications of PFAS include paper production, non-stick cookware, grease-resistant food packaging, stain-resistant furniture and carpets, waterproof textiles, cosmetics, and other fluorinated consumer products [13,14]. As a result, exposure to these substances occurs through diverse pathways (Figure 1). The primary route of human PFAS exposure is oral intake, mainly through contaminated food and drinking water. Seafood constitutes a particularly important source due to the persistence and bioaccumulation of PFAS in aquatic environments [14]. Other exposure routes include inhalation and dermal absorption [17]. Exposure can also occur in utero, and newborns may be exposed through breast milk [18]. Children may be particularly susceptible to PFAS exposure, as physiological and behavioral factors during early life can increase their body burden, resulting in higher serum concentrations than those observed in adults [14].

Although PFAS have been in use since the 1950s, concerns about their potentially harmful effects on human health only emerged in the early 2000s when significant amounts of perfluorooctanoic acid (PFOA) and perfluorooctanesulfonic acid (PFOS) were detected in human blood [9]. Current evidence indicates that PFAS exposure is most consistently associated with adverse immune effects, particularly in children, and dyslipidemia, while evidence for cancer remains limited to populations with exceptionally high occupational or environmental exposures, and the effects on neurodevelopment are still insufficiently characterized [19].

Recent evidence from alternative model systems, including *Drosophila melanogaster*, indicates that PFAS disrupt evolutionarily conserved signaling pathways involved in oxidative stress, metabolic homeostasis, and cellular stress responses, while also modulating pathways such as MAPK and PI3K/AKT and inducing compound-specific toxicological effects [20,21,22,23]. These findings support the concept that PFAS toxicity is mediated through complex alterations in gene regulatory and signaling networks.

Experimental studies in mammalian models have also demonstrated the genotoxic potential of PFOS. Chronic PFOS exposure in rats increased micronucleus frequency and strongly induced DNA damage in the liver, peripheral blood, and bone marrow [24,25,26]. In addition, PFOS induced oxidative damage and increased the expression of caspase-3 and caspase-8 in rat hepatocytes, indicating an apoptotic effect [26].

The accumulation of evidence regarding the adverse health effects of PFAS has prompted increasing regulatory and public health concern worldwide. As a result, the Stockholm Convention listed PFOS and its salts (PFOSF) in Annex B in 2009, while PFOA was added to Annex A in 2019, followed by PFHxS and its related compounds in 2022 [27,28]. In 2020, the European Food Safety Authority (EFSA) identified immune system effects, such as reduced antibody production in response to childhood vaccines and similar effects observed in animal experimental studies, as critical concerns [9]. Similarly, the U.S. Environmental Protection Agency has identified the immunotoxic effects of PFAS especially in children, as a key consideration in evaluating associated health risks [9].

However, the mechanisms underlying PFAS-induced immune effects remain incompletely understood, particularly regarding the molecular pathways, key gene targets, and immune processes involved, especially in the context of combined exposure.

**Figure 2:**
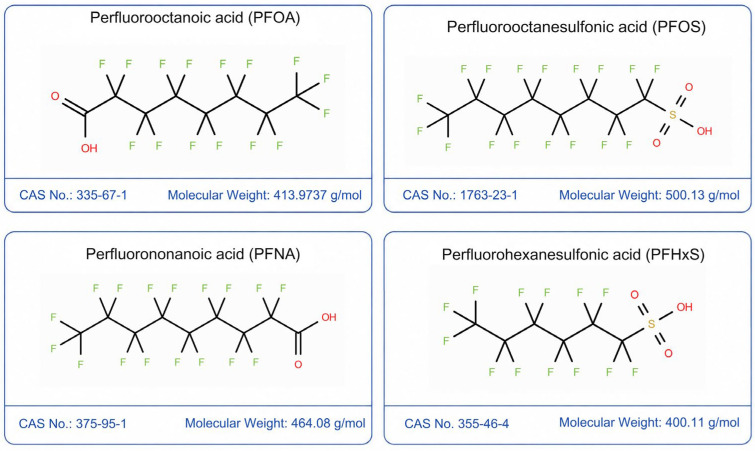
Chemical structures of the four PFAS compounds analysed in this study: perfluorooctanoic acid (PFOA), perfluorooctanesulfonic acid (PFOS), perfluorononanoic acid (PFNA), and perfluorohexanesulfonic acid (PFHxS).

The European Commission requested the EFSA to conduct a scientific risk assessment of the health effects of these substances in food. For this purpose, EFSA’s Contam Panel selected four PFAS compounds: PFOA, perfluorononanoic acid (PFNA), perfluorohexanesulfonic acid (PFHxS), and PFOS (Figure 2) [29]. These four compounds were chosen due to their significant presence in the environment, the availability of toxicokinetic data, and their substantial measured levels in human blood [29]. It is also important to note that exposure to other PFAS compounds, aside from the four mentioned above, primarily comes from substances with shorter elimination half-lives, which remain in the human body for a shorter duration [29,30].

As humans are continually exposed to a wide range of chemicals, assessing the risks of chemical mixtures has become increasingly important, while their combined effects may differ from those of individual substances [31,32]. Given the complexity of PFAS-induced biological effects and the large number of potential molecular targets involved, conventional experimental approaches alone are often insufficient to fully elucidate underlying mechanisms. As the number of newly discovered substances with potential toxicity to human health continues to rise, there is a growing demand for the development of more efficient in vitro and in silico models that could replace experimental studies on animals [33].

Driven by rapid technological advancements, toxicogenomics has advanced significantly, facilitating the identification of health risks by elucidating gene–environment interactions, including those arising from chemical mixtures, involved in disease development [34,35]. It is valuable for identifying gene ontology, which describes gene functions, biological processes, cellular components, and molecular pathways based on input gene sets. Additionally, it helps identify genomic biomarkers for further laboratory research and explore potential molecular mechanisms of toxicity [36,37]. It provides a framework for evaluating a wide range of interactions between chemicals and genes [34]. Within this context, network toxicology has emerged as a powerful systems-level approach, offering an integrative framework to understand how chemicals affect human health by mapping interactions across multiple layers of biological complexity [38].

Importantly, humans are not exposed to PFAS in isolation but rather to complex mixtures of environmental contaminants and food-related chemicals. Emerging evidence suggests that the biological effects of PFAS may be modified through interactions with other substances, including endocrine-disrupting chemicals and food additives such as artificial sweeteners, partly through shared effects on the gut microbiota and host metabolic and immune functions [39]. In addition, experimental studies have demonstrated that co-exposure to PFAS and mixtures of endocrine disruptors can potentiate developmental and endocrine-related effects [40], while recent mixture-entered approaches have highlighted the ability of PFAS-containing chemical mixtures to disrupt hormone-regulated and disease-relevant biological networks [41]. These observations further support the need for systems-level approaches to investigate the molecular mechanisms underlying PFAS-associated health effects.

Considering all of the above, the aim of the current study was to apply a network toxicology–based in silico approach to identify key gene targets, biological pathways, and molecular mechanisms underlying immune system disruption induced by a mixture of PFOA, PFNA, PFHxS, and PFOS. In the present study, the term “PFAS mixture” refers to the integrative analysis of molecular targets and biological pathways shared among selected representative PFAS compounds, rather than to experimentally determined biological responses following simultaneous exposure to a PFAS mixture.

## 2. Materials and Methods

**Figure 3:**
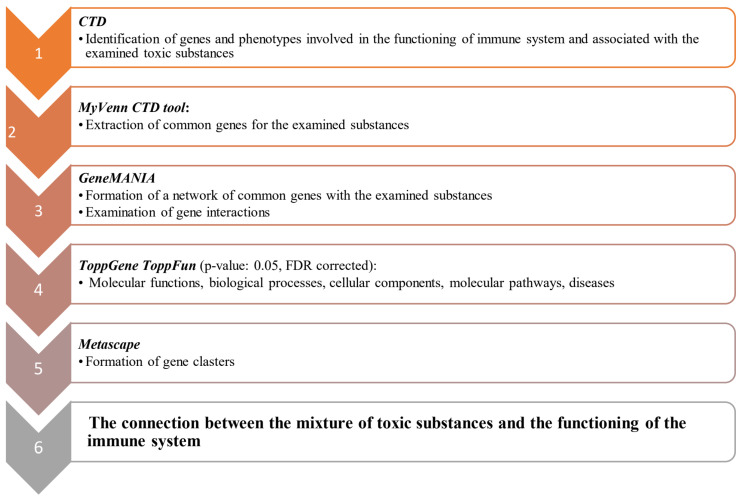
Flowchart illustrating the steps involved in the in silico analysis.

Due to their significant presence in the environment, available toxicokinetic data, and high concentrations measured in human blood [29], PFOA, PFNA, PFHxS, and PFOS were selected as substances of interest in this study. To analyze the impact of these substances on immune system function, the Comparative Toxicogenomics Database (CTD; MDI Biological Laboratory, Bar Harbor, ME, USA, and North Carolina State University, Raleigh, NC, USA; https://ctdbase.org, accessed on 3 April 2026 and 12 June 2026) was used as the primary tool for gene extraction and analysis. Additionally, the GeneMANIA server (University of Toronto, Donnelly Centre for Cellular and Biomolecular Research, Toronto, ON, Canada; https://genemania.org, accessed on 14 June 2026) and Cytoscape software (v3.10.4; Institute for Systems Biology, Seattle, WA, USA; https://cytoscape.org, accessed on 14 June 2026), including the GeneMANIA plugin (v3.5.3; https://apps.cytoscape.org/apps/genemania, accessed on 14 June 2026) and the CytoHubba plugin (v0.1; Department of Computer Science and Information Engineering, Nanhua University, Dalin Township, Chiayi County, Taiwan; https://apps.cytoscape.org/apps/cytohubba, accessed on 14 June 2026), as well as the ToppGene Suite portal (Computational Medicine Center, Cincinnati Children’s Hospital Medical Center, Cincinnati, OH, USA; https://toppgene.cchmc.org, accessed on 12 June 2026) and the Metascape platform (v3.5.20260201; https://metascape.org, accessed on 12 June 2026), were also utilized. A schematic overview of the in silico analysis pipeline employed in this study is shown in Figure 3**.** The present approach represents an in silico characterization of shared PFAS-associated molecular mechanisms and does not model direct experimental mixture effects.

### 2.1. Comparative Toxicogenomic Database (CTD)

The CTD (https://ctdbase.org) is a publicly available resource that integrates and organizes information on the relationships between chemicals, genes, and diseases. Additionally, it contains data on gene ontology (molecular functions, biological processes, cellular components), exposure to various chemicals and their mixtures, and phenotypic effects [42]. The database is regularly updated to ensure data accuracy, consistency, and availability [42,43,44,45]. In this study, CTD was used to identify genes associated with PFAS exposure and their impact on the immune system. All selected PFAS were successfully located in the CTD, and relevant genes linked to each of these substances and their effects on immunity were extracted. The genes were retrieved from the disease-associated sections of the database, considering all conditions related to immune function. For each PFAS, all available CTD chemical–gene interactions were retrieved without applying filters based on evidence type, interaction score, or species. Although the curated interactions included in the CTD are derived from studies conducted across multiple experimental species, the database is specifically designed to investigate the impact of environmental chemicals on human health and therefore includes only genes and proteins with human orthologs [46]. To determine the genes common to all four analysed PFAS, the MyVenn tool available within the CTD was used. Immune system–related phenotypes were extracted from CTD phenotype data cards; such data were available for PFOA, PFOS, and PFHxS, but not for PFNA. Information on phenotype, interaction, organisms, anatomy, and references was retrieved directly from CTD, while study design details were obtained from the corresponding referenced literature cited in CTD.

### 2.2. GeneMANIA and CytoHubba

GeneMANIA (https://genemania.org) identifies multiple types of gene interactions, including physical interactions (protein–protein binding), co-expression (similar expression patterns), genetic interactions (functional associations), shared protein domains, co-localization (activity within the same tissue or cellular compartment), and involvement in common molecular pathways [47]. In this study, the GeneMANIA server was used to analyse interactions among the identified genes, focusing on the human organism (*H. sapiens*), particularly using the Cytoscape plug-in version [48]. Subsequently, the CytoHubba tool within Cytoscape software (v3.10.4) was employed to identify and rank the ten most significant genes within the constructed GeneMANIA network. Hub genes were ranked using the Maximum Clique Centrality (MCC) algorithm implemented in the CytoHubba plugin of Cytoscape. The MCC method was selected because it has been reported to outperform several alternative topological algorithms in identifying biologically important network nodes, demonstrating superior precision for the prediction of essential proteins in protein–protein interaction networks [49].

### 2.3. ToppGene Suite Portal

The ToppGene Suite portal (https://toppgene.cchmc.org) provides tools for analyzing the biological functions of genes. Its ToppFun module allows comprehensive exploration of gene ontology, molecular pathways, phenotypes, microRNA interactions, and additional functional annotations [50]. In this study, ToppFun was used to analyse potential molecular mechanisms associated with the effects of PFAS mixtures on the immune system. A set of genes common to all four investigated PFASs was examined, focusing on molecular functions, biological processes, cellular components, pathways, and diseases. The significance of the results was assessed based on a *p*-value ≤ 0.05 with FDR correction.

### 2.4. Metascape

Metascape (https://metascape.org) is an online platform for the annotation and analysis of gene sets, while the MCODE (Molecular Complex Detection) tool identifies densely connected regions within gene networks, i.e., gene clusters. In this study, MCODE was used to detect clusters within the network of genes shared by all four PFAS and associated with immune system effects. The resulting MCODE network highlights groups of genes that physically interact with at least one other gene in the input set, making it particularly useful for identifying highly connected network components [51].

## 3. Results

**Figure 4:**
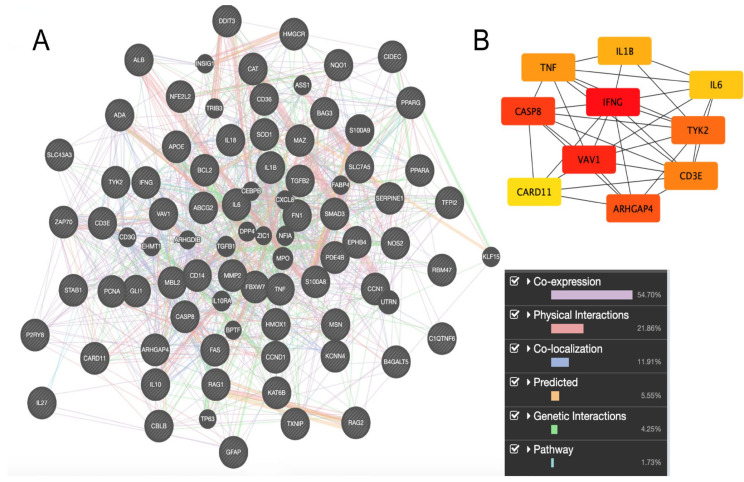
(**A**). Interactions between genes associated with exposure to PFAS mixture (GeneMANIA: https://genemania.org, accessed on 14 June 2026); (**B**). The 10 identified most significant genes (CytoHubba Cytoscape; https://apps.cytoscape.org/apps/cytohubba, accessed on 14 June 2026). Node colors represent gene significance in the network based on the selected centrality testing method (MCC—Maximum Clique Centrality). Genes with the highest significance are marked in red, genes with moderate significance in orange, and genes with the lowest significance in yellow.

A total of 707, 568, 235, and 223 genes associated with the immune system and exposure to PFOS, PFOA, PFHxS, and PFNA, respectively, were extracted. Of these genes, 65 were common to all four studied PFAS: *ABCG2*, *ADA*, *ALB*, *APOE*, *ARHGAP4*, *B4GALT5*, *BAG3*, *BCL2*, *C1QTNF6*, *CARD11*, *CASP8*, *CAT*, *CBLB*, *CCN1*, *CCND1*, *CD14*, CD36, CD3E, *CIDEC*, *DDIT3*, *EPHB4*, *FAS*, *FBXW7*, *FN1*, *GFAP*, *GLI1*, *HMGCR*, *HMOX1*, *IFNG*, *IL10*, *IL18*, *IL1B*, *IL27*, *IL6*, *KAT6B*, *KCNN4*, *MAZ*, *MBL2*, *MMP2*, *MSN*, *NFE2L2*, *NOS2*, *NQO1*, *P2RY8*, *PCNA*, *PDE4B*, *PPARA*, *PPARG*, *RAG1*, *RAG2*, *RBM47*, *S100A8*, *SERPINE1*, *SLC43A3*, *SLC7A5*, *SMAD3*, *SOD1*, *STAB1*, *TFPI2*, *TGFB2*, *TNF*, *TXNIP*, *TYK2*, *VAV1*, and *ZAP70*. More than half of these genes were co-expressed (54.70%), while the second most significant form of gene interaction was physical interaction (21.86%) (Figure 4A). From the constructed gene interaction network, the top 10 hub genes, identified as the most highly connected and functionally central nodes, were selected: *IFNG*, *VAV1*, *CASP8*, *ARHGAP4*, *TYK2*, *CD3E*, *TNF*, *IL1B*, *IL6*, and *CARD11*. Of these 10 most significant genes, *IFNG* and *VAV1* were identified as key genes in interactions and regulation of biological processes. *CASP8*, *ARHGAP4*, *TYK2*, *CD3E*, and *TNF* were identified as genes of moderate significance, while *IL1B*, *IL6*, and *CARD11* were identified as genes of lower significance (Figure 4B).

Table 1 summarizes the interactions between PFAS and key immune-related genes, presenting effects at both the mRNA and protein expression levels. For each gene, the table indicates how different PFAS influence expression, showing whether they cause upregulation or downregulation. The symbols used denote the direction of these effects: “+” indicates increased expression, “−” indicates decreased expression, “+/−” indicates that both increased and decreased expression have been reported depending on the study design from which the interaction was derived, and “NA” indicates that no data are available in the CTD.

Table 2 presents immune-related phenotypes associated with the investigated PFAS extracted from the CTD, including information on chemical–phenotype interactions, organisms, and anatomical sites. No immune-related phenotypes were identified for PFNA.

For PFOA, data were available for *Mus musculus* and *Homo sapiens*. In mice, PFOA was associated with the immune response in the spleen and lymph nodes, including decreased B cell proliferation, increased monocyte proliferation, and both increased and decreased CD4^+^ and CD8^+^ T cell proliferation, depending on the tissue (spleen or lymph nodes). Additional phenotypes in mice include increased immunoglobulin production in blood. In humans, PFOA was linked to decreased adaptive immune response and decreased activation of immune response in plasma, as well as decreased humoral immune response in serum.

For PFOS, phenotypes were also reported in *Mus musculus* and *Homo sapiens*. In mice, PFOS is linked to increased positive regulation of innate immune response in the liver, lung, and kidney. In humans, PFOS is associated with decreased humoral immune response, decreased circulating IgG immunoglobulin complex, decreased overall immune response, and altered immunoglobulin production, all identified in serum.

For PFHxS, only human data are reported, showing decreased humoral immune response and decreased circulating IgG immunoglobulin complex in serum.

Table 3 presents the results of enrichment analysis for genes common to all four analyzed PFAS, grouped into five categories: molecular functions, biological processes, cellular components, molecular pathways, and diseases. Within each category, the ten most significantly enriched terms were selected and ranked according to their *p*-values, with lower *p*-values indicating stronger statistical significance. For each enriched term, the corresponding interacting genes contributing to the enrichment are also provided.

**Figure 5:**
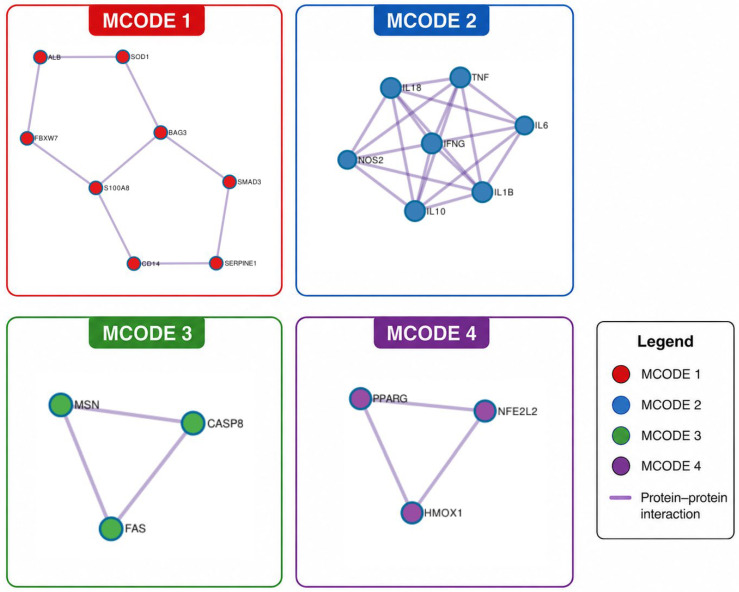
Interconnected gene clusters involved in the toxicity mechanism of PFAS chemicals obtained through MCODE analysis (Metascape software; https://metascape.org, accessed on 12 June 2026).

The networks generated using MCODE analysis are shown in Figure 5 and Table 4. Four interconnected gene clusters were identified. The first cluster was primarily associated with vitamin B12 and folate metabolism, together with the positive regulation of the intrinsic apoptotic signaling pathway. The second cluster was enriched for pathways related to post-COVID neuroinflammation, integrative analysis of regulatory T cell (Treg)–glial interactions, and the PID IL23 signaling pathway. The third cluster was linked to measles and measles virus infection, while the fourth was associated with the regulation of endothelial cell migration and angiogenesis. In addition, the overall network enrichment analysis highlighted signaling by interleukins, cytokine signaling in the immune system, and interleukin-4/interleukin-13 signaling as the most significantly enriched pathways.

## 4. Discussion

In previously published studies, the impact of PFAS mixtures on reduced activation of immune cells, such as CD4 and CD8 T lymphocytes, which are key players in the adaptive immune response, has been identified. The effect of PFHxS, PFOA, PFOS, PFNA, perfluorobutanoic acid (PFBa), and perfluorohexanoic acid (PFHxA) were examined on the activation of primary human immune cells in vitro. The results showed that exposure to these substances reduced T cell activation, affecting T helper cells, cytotoxic T cells, and MAIT (Mucosal-associated invariant T) cells, as well as gene and protein expression important for their function. The authors also demonstrated that the mixture of tested PFAS had the most pronounced effect compared to the individual substances [61]. Another in vitro study investigated the sensitivity of immune cells to various PFAS (including PFOS and PFOA), comparing Jurkat T-cells and TNR-1 monocytes. The results showed that monocytes were more resistant, but PFAS caused their necrosis, which could contribute to inflammatory effects [62]. Additionally, the effect of different PFAS mixtures (a binary mixture of PFOS and PFOA (1.88 mg PFOS + 1.88 mg PFOA/kg body mass/day) and a quaternary mixture of PFOS, PFOA, PFHxS, and PFNA (1.88 mg PFOS + 1.88 mg PFOA + 0.376 mg PFHxS + 0.26 mg PFNA/kg body mass/day) on a mouse influenza infection model was examined. It was found that the binary mixture affected T-cell immunity, while the quaternary mixture affected B-cell immunity, indicating that the immunomodulatory effects of PFAS depend on the cell type and mixture composition [63]. The effect of PFAS (PFOA, PFOS, PFNA, and PFHxS) on the immune cell profile was also investigated in humans, in a study involving 50 participants. The results showed that PFAS were associated with an increased frequency of NK/T cells and activated Th memory cells, as well as a decrease in the frequency of Tc cells with CXCR3+ effector memory phenotype, indicating potential immune-modulating mechanisms [64]. Furthermore, another human study examined the effects of these substances on the immune system by analyzing changes in genomic transcriptomes in peripheral blood mononuclear cells (PBMCs) in adults from the Czech Republic. It was discovered that exposure to different PFAS disrupted transcriptomic networks related to adaptive immunity, particularly affecting processes involved in the later development of B lymphocytes, such as B cell receptor signaling and plasma cell development. The study identified disrupted B cell maturation as a potential mechanism of immunotoxicity for these substances, highlighting their effect on antibody production [65]. These studies suggest that PFAS exposure does not induce a uniform immunological outcome, but rather results in context-dependent immune modulation involving both suppressive and pro-inflammatory responses across different immune cell populations and experimental systems.

Despite growing evidence from experimental and epidemiological studies, the molecular mechanisms underlying PFAS-induced immunotoxicity remain incompletely characterized, particularly in the context of combined exposure to multiple PFAS compounds. In particular, there is a lack of integrative analyses identifying shared gene networks and central regulatory hubs affected by multiple PFAS simultaneously. Therefore, the aim of this study was to further investigate the mechanisms and potential consequences of exposure to a mixture of PFOA, PFNA, PFHxS, and PFOS on immune system function by identifying common gene networks affected by the PFAS mixture and predicting their functional consequences. Sixty-five genes common to all four tested substances were identified. Identification of shared genes across all the tested substances suggests convergence toward common immunotoxic pathways, despite structural differences among PFAS compounds. More than half of these genes were involved in co-expression (54.70%), while the second most significant form of gene interaction was physical interaction (21.86%). Co-expression suggests potential common pathways for gene action, while physical interactions indicate their direct collaboration in affecting the immune system. *IFNG*, *VAV1*, *CASP8*, *ARHGAP4*, *TYK2*, *CD3E*, *TNF*, *IL1B*, *IL6*, and *CARD11* were identified as the most significant genes, and thus representing potential candidate biomarkers for assessing PFAS-associated immunotoxic effects. *VAV1*, *ARHGAP4*, and *CARD11* are involved in signaling and the regulation of the cytoskeleton. *VAV1* serves as a hematopoietic-specific guanine nucleotide exchange factor (GEF) that mainly promotes RAC activation in T lymphocytes, while *CARD11* functions as a scaffold component of the CBM complex, enabling antigen receptor-triggered NF-κB signaling through TCR- and CD28-associated pathways in both T and B cells [66]. *ARHGAP4* encodes a Rho GTPase-activating protein (RhoGAP) that regulates members of the Rho family of small GTPases, particularly those involved in remodeling of the actin cytoskeleton [67]. *CASP8* encodes caspase-8, an initiator caspase that plays a key role in apoptosis, a programmed cell death process essential for mammalian development and immune function [68]. IL-1β and TNF-α act as early pro-inflammatory mediators that induce vascular activation, leukocyte recruitment, and fever, while IL-6 supports acute-phase responses and connects innate and adaptive immunity, and IFN-γ enhances antimicrobial immune responses through macrophage activation and increased antigen presentation [69]. *TYK2* encodes tyrosine kinase 2, a signaling kinase that associates with receptor components for several immune cytokines, including type I interferons and interleukins such as IL-6, IL-10, IL-12, and IL-23 [70]. CD3E encodes the CD3 epsilon chain, a key component of the CD3 complex, which forms part of the T cell receptor (TCR) signaling machinery and provides the initial activation signal upon antigen recognition by T lymphocytes [71,72]. Each of these genes contributes to distinct aspects of metabolism and function in the organism, which could potentially be disrupted by PFAS exposure. The combined effects of these molecules suggest that their damage or dysfunction could weaken the organism’s ability to fight infections and increase the risk of inflammatory and autoimmune diseases, thereby disrupting the immune response balance. The results of interaction analysis between the chemicals and genes indicate that some key genes are particularly sensitive to the effects of all tested PFAS. For example, *ARHGAP4*, *CD3E*, and *CARD11* show reduced expression at the RNA level under the action of all tested substances, which may indicate the existence of a common toxicity mechanism. A reduction in *TYK2* RNA expression was also observed as a consequence of PFAS exposure, with dual effects (+/−) noted for PFOS. On the other hand, RNA or protein expression of the *IL1B* gene was increased under the influence of all tested substances, indicating its activation. A similar pattern of increased RNA or protein expression was observed for *IL6*, where the majority of the investigated PFAS led to increased expression.

To further contextualize the gene-based findings, immune-related phenotype data were extracted from the CTD. These data provide curated links, supported by experimental and epidemiological evidence, between PFAS exposure and functional immune outcomes. However, as CTD phenotype entries are presented in a condensed format, additional details regarding study design, exposure conditions, doses, and measured endpoints were retrieved from the original publications referenced within CTD. This approach was applied to ensure accurate interpretation of the reported interactions, enable comparison across experimental and human studies, and provide a more comprehensive understanding of the biological relevance, dose-dependency, and translational significance of PFAS-induced immunotoxic effects. The compiled CTD phenotype data provide consistent evidence that PFAS exposure targets key components of adaptive and humoral immunity, while also inducing context-dependent activation of innate immune responses across species. For PFOA, experimental findings in *Mus musculus* demonstrate pronounced immunosuppressive effects following dermal exposure at 0.5–2% (*w*/*v*) (approximately 12.5–50 mg/kg/dose) [52]. These include reduced IgM responses, decreased spleen and thymus weight, and diminished splenic B-cell populations, indicating impaired humoral immunity, likely mediated via PPARα activation. At the same time, PFOA induced tissue-specific alterations in immune cell proliferation, with increased CD4^+^ and CD8^+^ T-cell proliferation in the spleen, but decreased proliferation in lymph nodes, alongside increased monocyte proliferation [52]. Additional experimental data in mice exposed to higher doses (100–150 mg/kg) showed enhanced mast cell activation, increased intracellular Ca^2+^, elevated pro-inflammatory cytokines (TNF-α, IL-1β, IL-6, IL-8), and increased immunoglobulin production, suggesting that high-dose exposure may shift immune responses toward hypersensitivity and inflammation [56]. Human data (*Homo sapiens*) strongly support the immunosuppressive effects of PFOA at environmentally relevant exposure levels. In a cross-sectional study of 1-year-old children, plasma concentrations ranged from approximately 3.8 ± 1.1 µg/L in formula-fed infants to 16.8 ± 6.6 µg/L in breastfed infants, and were associated with decreased activation of the immune response and reduced adaptive immunity [53]. More robustly, prospective birth cohort studies demonstrated that a twofold increase in serum PFAS exposure (including PFOA) resulted in reductions in vaccine-induced antibody levels, clearly indicating impaired humoral immune function [54,55]. For PFOS, similar immunotoxic patterns were observed. Experimental studies in mice demonstrated that PFOS can activate innate immune pathways, specifically through activation of the AIM2 inflammasome, leading to increased IL-1β release, pyroptosis, and inflammation in liver, lung, and kidney tissues [60]. In humans, serum PFOS exposure across multiple cohorts (children and adolescents) was consistently associated with decreased circulating IgG levels, reduced humoral immune responses, and diminished vaccine antibody production [54,57,58,59,73]. For PFHxS, although data were more limited, human epidemiological studies indicate decreased humoral immune responses at typical exposure levels measured in serum during childhood, particularly when combined with other PFAS. These findings include reduced antibody responses to vaccines, as well as decreased circulating IgG levels into adolescence [54,55].

To bridge the observed PFAS-associated immune phenotypes with their molecular mechanisms, MCODE analysis was performed on the constructed gene interaction network to identify highly interconnected functional modules driving the reported immunotoxic effects. MCODE analysis identified four interconnected gene clusters. The first cluster was enriched for pathways related to vitamin B12 metabolism, folate metabolism, and the positive regulation of the intrinsic apoptotic signaling pathway. The enrichment of apoptosis-related processes is consistent with previous reports indicating that PFAS exposure may induce oxidative stress and apoptotic responses [74,75], supporting the involvement of these mechanisms in PFAS-associated toxicity. The second cluster was associated with post-COVID neuroinflammation, integrative analysis of regulatory T cell (Treg)–glial interactions, and the PID IL23 pathway. These findings likely reflect shared molecular signatures and should not be interpreted as direct evidence that PFAS exposure causes or exacerbates these disease conditions. Rather, they point toward the involvement of immune-regulatory and inflammatory pathways that overlap with those activated in various pathological states. The third cluster included pathways related to measles and measles virus infection, again suggesting convergence on common immune signaling mechanisms rather than a causal relationship between PFAS exposure and infectious diseases. The fourth cluster was associated with the regulation of endothelial cell migration and angiogenesis, indicating that PFAS may influence processes involved in vascular remodeling and tissue homeostasis. This interpretation is supported by recent findings demonstrating that PFOS promotes vascular cell migration and phenotypic switching, thereby contributing to vascular remodeling and altered tissue homeostasis [76]. Notably, this same MCODE cluster comprised *PPARG*, *NFE2L2*, and *HMOX1*. Although *PPARG* was not prioritized among the highest-ranked hub genes, its inclusion within a highly interconnected functional module suggests that it may contribute to PFAS-associated molecular responses through interactions with other functionally related genes. This observation is consistent with previous experimental evidence implicating PPARγ signaling in PFAS-induced inflammatory and metabolic alterations [77,78]. It has also been suggested that PFAS-induced upregulation of the pro-inflammatory cytokines IL-6 and IL-8 is mediated, at least in part, through PPARγ signaling [79]. Interestingly, the co-occurrence of *PPARG*, *NFE2L2*, and *HMOX1* within the same functional module is supported by evidence that PPARγ cooperates with Nrf2 to regulate HMOX1 expression, thereby linking inflammatory and antioxidant signaling pathways [80]. In addition to these MCODE-derived modules, the overall network enrichment analysis highlighted signaling by interleukins, cytokine signaling in the immune system, and interleukin-4/interleukin-13 signaling as the most significantly enriched pathways. IL-4 and IL-13 are key regulators of allergic inflammation that influence lymphoid, myeloid, and non-hematopoietic cells. They promote Th2 differentiation in CD^4+^ T cells, drive IgG1 and IgE class switching in B cells, and induce alternative activation of macrophages [80].

To complement the MCODE-derived network modules and provide a more comprehensive functional interpretation of the identified gene clusters, Gene Ontology (GO) enrichment analysis was performed. Molecular functions, biological processes, cellular components, molecular pathways, and diseases associated with genes common to all tested PFAS are numerous and indicate their significant impact on immune process modulation. Among the molecular functions, activities such as binding to signaling receptors, cytokine activity, and antioxidant activity, as well as binding to fatty acids, phosphatases, and proteases, stand out, suggesting that they affect key regulators of the immune response. Biological processes such as apoptosis regulation, cytokine production, immune response regulation, and cell activation clearly point to the ability of these compounds to affect the balance between pro- and anti-inflammatory mechanisms. Additionally, cellular components like membrane microdomains, platelet alpha-granules, and the outer side of the plasma membrane suggest potential effects on the plasma membrane and signaling complexes of immune cells, which could alter their function. Moreover, signaling pathways such as IL-4/IL-13 signaling, other cytokine signaling pathways, and the AP1 survival signaling pathway further demonstrate the complex molecular mechanisms through which the tested mixture may modulate immune activity. Finally, these pathways overlap with molecular signatures previously implicated in various autoimmune diseases (rheumatoid arthritis and asthma) as well as metabolic and cardiovascular disorders (experimental diabetes and hypertensive disease). These associations should be interpreted as shared biological mechanisms rather than direct evidence that PFAS exposure causes or exacerbates these disease outcomes.

The utility of network toxicology and integrated bioinformatics approaches has been demonstrated for a variety of environmental contaminants beyond PFAS, including heavy metals [81], endocrine-disrupting chemicals [82,83,84,85], and other environmental pollutants and complex chemical mixtures [86,87], as well as for identifying potential protective agents [88,89]. In these studies, computational predictions have successfully highlighted pathways related to oxidative stress, inflammation, immune dysregulation, and endocrine signaling, many of which were subsequently supported by in vitro, in vivo, and epidemiological evidence. The consistency of these findings across different classes of toxicants provides additional support for the biological plausibility of the pathways and hub genes identified in the present analysis and supports the use of systems-level approaches as valuable NAMs for hypothesis generation and target prioritization.

The current study provides a valuable systems-level framework for identifying shared molecular mechanisms potentially underlying PFAS mixture-induced immunotoxicity. It is particularly useful for prioritizing candidate biomarkers and immune-related pathways relevant to cumulative exposure assessment and for generating hypotheses for future experimental studies. The findings should be interpreted as reflecting convergent biological pathways and molecular signatures associated with multiple PFAS compounds, rather than experimentally validated effects of simultaneous PFAS exposure. Nevertheless, the identification of these common targets may help elucidate mechanisms that contribute to the immunotoxic potential of real-world PFAS co-exposures and guide future in vitro and in vivo investigations. An additional area that warrants further investigation is the potential interaction between PFAS and other persistent emerging contaminants, including artificial sweeteners such as sucralose. Recent studies have highlighted the structural similarity between sucralose and halogenated compounds such as PFAS and have suggested that both may be regarded as persistent environmental contaminants with widespread occurrence, making co-exposure a plausible real-world scenario that should be addressed in future mixture toxicity studies [90,91].

Nevertheless, several limitations should be acknowledged. Gene network analysis is based on publicly available databases and in silico predictions, which depend on the completeness of curated interaction data and may not capture all PFAS–gene interactions. CTD phenotype data are heterogeneous in species, exposure routes, doses, and experimental designs, limiting direct comparability and translational extrapolation. In addition, network analyses identify potential regulatory hubs but do not confirm causality, so proposed biomarkers should be considered predictive and require experimental validation.

Similarly, functional enrichment analyses identify biological pathways and disease-associated molecular signatures that overlap with the analysed gene set, but they do not establish direct causal relationships between PFAS exposure and specific disease outcomes. In addition, systems-level computational analyses are inherently dependent on the content and curation status of the underlying databases. Consequently, the identification and ranking of hub genes and enriched modules may evolve as new biological interaction data become available.

## 5. Conclusions

A total of 65 genes common to all four PFAS (PFOA, PFOS, PFNA and PFHxS) were identified, with co-expression as the dominant interaction type followed by physical interactions. Key hub genes (*IFNG*, *VAV1*, *CASP8*, *ARHGAP4*, *TYK2*, *CD3E*, *TNF*, *IL1B*, *IL6*, and *CARD11*) indicate disruption of cytokine signaling, T- and B-cell activation, apoptosis, and cytoskeletal regulation, suggesting impaired immune homeostasis and increased susceptibility to inflammatory and autoimmune responses. Chemical–gene interaction patterns showed consistent downregulation of *ARHGAP4*, *CD3E*, and *CARD11*, alongside upregulation of *IL1B* and *IL6*, indicating shared toxicity mechanisms and possible additive or synergistic effects. Network clustering revealed four major functional modules associated with (i) vitamin B12 and folate metabolism together with the intrinsic apoptotic signaling pathway, (ii) immune and inflammatory processes related to post-COVID neuroinflammation, Treg–glial interactions, and IL-23 signaling, (iii) shared molecular signatures of viral infection pathways, and (iv) endothelial cell migration and angiogenesis. In addition, global network enrichment highlighted IL-4/IL-13-mediated cytokine signaling involved in Th2 differentiation, immunoglobulin class switching, and allergic inflammation. Functional enrichment further supported broad immune modulation, highlighting cytokine activity, immune cell activation, apoptosis regulation, and key signaling pathways including IL-4/IL-13 and AP-1. Phenotype-level evidence confirmed immune dysregulation, showing decreased humoral and adaptive immune responses in humans and tissue-specific modulation of B- and T-cell activity in mice, while no immune-related phenotypes were observed for PFNA. These results indicate that PFAS collectively target central immune regulatory networks involved in inflammation, immune balance, and disease susceptibility. The present findings provide a systems-level framework for understanding the shared immunotoxic mechanisms of representative PFAS and may contribute to the identification of candidate biomarkers and pathways relevant to real-world co-exposure scenarios. The computational approach presented here may also support future hazard assessment and the prioritization of targets for experimental validation within modern NAMs.

## Figures and Tables

**Table 1:** Interactions between PFASs and key genes associated with the immune system.

Gene	Interaction	*PFOA*	*PFOS*	*PFNA*	*PFHxS*
*IFNG*	ME	+	NA	NA	NA
	PE	NA	−	−	NA
*VAV1*	ME	+	NA	NA	+
	PE	NA	NA	NA	NA
*CASP8*	ME	NA	−	NA	+
	PE	NA	NA	−	NA
*ARHGAP4*	ME	−	−	−	−
	PE	NA	NA	NA	NA
*TYK2*	ME	+/−	−	−	−
	PE	NA	NA	NA	NA
*CD3E*	ME	−	−	−	−
	PE	NA	NA	NA	NA
*TNF*	ME	+	+/−	NA	−
	PE	+	+/−	+	NA
*IL1B*	ME	+	+/−	NA	+
	PE	+	+	+	NA
*IL6*	ME	+	+	NA	+
	PE	+	NA	+	NA
*CARD11*	ME	−	−	−	−
	PE	NA	NA	NA	NA

ME—mRNA expression; PE—protein expression; NA—not available. +—increased expression; −—decreased expression; +/−—expression can be both decreased and increased, depending on the study design from which the interaction was sourced.

**Table 2:** Immune-related phenotypes associated with selected PFAS (PFOA, PFOS, and PFHxS) extracted from the Comparative Toxicogenomics Database (CTD; https://ctdbase.org, accessed on 3 April 2026 and 12 June 2026).

Chemical	Phenotype	Interaction	Organisms	Anatomy	Study Design	CTD ID References
PFOA	immune response	PFOA affects immune response	*Mus musculus*	Spleen	Study type: Experimental murine study Exposure: Dermal PFOA (0.5–2% *w*/*v*; 12.5–50 mg/kg/dose) Key findings: Dermal PFOA reduced IgM response, decreased thymus and spleen weight, increased liver weight, reduced splenic B-cells; involvement of PPARα	31904477 [52]
		[PFOA affects immune response] which results in decreased B cell proliferation	*Mus musculus*	Spleen|B-Lymphocytes		
		[PFOA affects immune response] which results in increased monocyte proliferation	*Mus musculus*	Spleen|Monocytes		
		[PFOA affects immune response] which results in increased T cell proliferation	*Mus musculus*	Spleen|CD4-Positive T-Lymphocytes		
		[PFOA affects immune response] which results in increased T cell proliferation	*Mus musculus*	Spleen|CD8-Positive T-Lymphocytes		
		[PFOA affects immune response] which results in decreased T cell proliferation	*Mus musculus*	Lymph Nodes|CD4-Positive T-Lymphocytes		
		[PFOA affects immune response] which results in decreased T cell proliferation	*Mus musculus*	Lymph Nodes|CD8-Positive T-Lymphocytes		
PFOA	adaptive immune response	PFOA results in decreased adaptive immune response	*Homo sapiens*	Plasma	Study type: Cross-sectional study Population: 101 healthy 1-year-old children Exposure: Plasma PFAS levels (PFOA, PFOS); formula-fed vs. breastfed Measured concentrations (mean ± SD): Formula-fed: PFOA 3.8 ± 1.1 µg/L, PFOS 6.8 ± 3.4 µg/L Breastfed: PFOA 16.8 ± 6.6 µg/L, PFOS 15.2 ± 6.9 µg/L	32227269 [53]
PFOA	humoral immune response mediated by circulating immunoglobulin	PFOA results in decreased humoral immune response mediated by circulating immunoglobulin	*Homo sapiens*	Serum	22274686: Study type: Prospective birth cohort (Faroe Islands, 1997–2000; 587 followed) Exposure: Serum PFOS, PFOA in mothers and children at age 5 Endpoints: Tetanus and diphtheria antibody levels at ages 5 and 7 Key findings: Higher PFAS → lower antibody levels; 2× exposure associated with −39 to −49% antibody reduction and increased odds of falling below protective levels 26041029: Study type: Prospective birth cohort (Faroese cohort, 464 children) Exposure: Serum PFAS concentrations at ages 5 and 7 Endpoints: Diphtheria and tetanus antibody levels at ages 5 and 7 Key findings: 2× higher PFAS → ~54% decrease in antibody levels; combined age-5 and age-7 exposures slightly greater reductions	22274686|26041029 [54,55]
PFOA	immunoglobulin production involved in immunoglobulin-mediated immune response	PFOA results in increased immunoglobulin production involved in immunoglobulin-mediated immune response	*Mus musculus*	Blood	Study type: Experimental (in vitro mast cells + in vivo mice) Exposure: PFOA, 100–150 mg/kg, given 3 times, on days 9, 11, 13. Endpoints: Mast cell degranulation, intracellular Ca^2+^, pro-inflammatory cytokines (TNF-α, IL-1β, IL-6, IL-8), allergic responses (hypothermia, histamine, TNF-α, IgE, IgG) Findings: PFOA enhanced mast cell degranulation, upregulated cytokines, and worsened allergic responses	27682001 [56]
		PFOA promotes the reaction [OVAL protein results in increased immunoglobulin production involved in immunoglobulin-mediated immune response]	*Mus musculus*	Blood		
PFOA	T cell proliferation involved in immune response	PFOA results in increased T cell proliferation involved in immune response	*Mus musculus*	Spleen|CD4-Positive T-Lymphocytes	Study type: Experimental murine study Exposure: Dermal PFOA (0.5–2% *w*/*v*; 12.5–50 mg/kg/dose) Key findings: Dermal PFOA reduced IgM response, decreased thymus and spleen weight, increased liver weight, reduced splenic B-cells; involvement of PPARα	31904477 [52]
		PFOA results in increased T cell proliferation involved in immune response	*Mus musculus*	Spleen|CD8-Positive T-Lymphocytes		
		PFOA results in decreased T cell proliferation involved in immune response	*Mus musculus*	Lymph Nodes|CD4-Positive T-Lymphocytes		
		PFOA results in decreased T cell proliferation involved in immune response	*Mus musculus*	Lymph Nodes|CD8-Positive T-Lymphocytes		
PFOA	activation of immune response	PFOA results in decreased activation of immune response	*Homo sapiens*	Plasma	Study type: Cross-sectional study Population: 101 healthy 1-year-old children Exposure: Plasma PFAS levels (PFOA, PFOS); formula-fed vs. breastfed Measured concentrations (mean ± SD): Formula-fed: PFOA 3.8 ± 1.1 µg/L, PFOS 6.8 ± 3.4 µg/L Breastfed: PFOA 16.8 ± 6.6 µg/L, PFOS 15.2 ± 6.9 µg/L	32227269 [53]
PFOS	humoral immune response mediated by circulating immunoglobulin	PFOS results in decreased humoral immune response mediated by circulating immunoglobulin	*Homo sapiens*	Serum	22274686: Study type: Prospective birth cohort (Faroe Islands; 656 recruited, 587 followed) Exposure: Serum PFOS, PFOA in maternal pregnancy samples and child serum at age 5 Measured concentrations: PFOS and PFOA were the dominant PFAS Endpoints: Tetanus and diphtheria antibody levels at ages 5 and 7; Key findings: 2× higher PFAS exposure → −39% to −49% antibody levels and increased risk (OR 2.38–4.20) of falling below protective levels 26041029: Study type: Prospective birth cohort (Faroese cohort; 464 children) Exposure: Serum PFASs at ages 5 and 7 Measured concentrations: PFAS levels quantified in serum at both time points Endpoints: Diphtheria and tetanus antibody levels Key findings: 2× higher combined PFAS exposure → ~54% decrease in antibody levels; stronger effect when considering both age-5 and age-7 exposures	22274686|26041029 [54,55]
PFOS	IgG immunoglobulin complex, circulating	PFOS results in decreased IgG immunoglobulin complex, circulating	*Homo sapiens*	Serum	Study type: Prospective birth cohort follow-up (Faroese cohort; 516 adolescents, age 13) Exposure: Serum PFASs measured at ages 7 and 13 Measured concentrations: PFAS levels quantified in serum at both time points Endpoints: Diphtheria and tetanus antibody levels (age 13 vs. age 7) Key findings: Higher PFAS exposure → ~10–30% (SEM) and ~25% (regression) reduction in diphtheria antibodies per 2× exposure; limited effects on tetanus antibodies	27501995 [57]
PFOS	immune response	PFOS results in decreased immune response	*Homo sapiens*	Serum	Study type: Randomized controlled trial with prospective follow-up (subset analysis; *n* = 237) Population: Children from Guinea-Bissau, followed from 4–7 months to 2 years Exposure: Serum PFAS (six compounds) measured at inclusion Measured concentrations: All PFASs detectable in nearly all children; levels lower than in high-income settings Endpoints: Measles antibody concentrations (pre- and post-vaccination) Infant morbidity (maternal reports) Key findings: 2× higher PFOS and PFDA → 21% and 25% lower measles antibody levels at 9 months Higher PFAS levels associated with lower pre-vaccination antibodies and increased morbidity	32772733 [58]
PFOS	immunoglobulin production involved in immunoglobulin-mediated immune response	PFOS affects immunoglobulin production involved in immunoglobulin-mediated immune response	*Homo sapiens*	Serum	Study type: Prospective cohort (n = 411 adults) Exposure: Serum PFOA and PFOS Measured concentrations (median): PFOA 31.5 ng/mL, PFOS 9.2 ng/mL Endpoints: Influenza vaccine antibody response and protective threshold Key findings: Higher PFOA → reduced antibody response and lower likelihood of protective levels; no effect for PFOS	24284791 [59]
PFOS	positive regulation of innate immune response	PFOS results in increased positive regulation of innate immune response	*Mus musculus*	Liver	Study type: Experimental in vitro and in vivo (AIM2 knockout mice) Exposure: PFOS (concentration not specified) Endpoints: AIM2 inflammasome activation, IL-1β release, pyroptosis, tissue inflammation (lung, liver, kidney) Key findings: PFOS triggers AIM2 → inflammation and tissue damage; reduced effects in Aim2^−^/^−^ mice	34006824 [60]
		PFOS results in increased positive regulation of innate immune response	*Mus musculus*	Lung		
		PFOS results in increased positive regulation of innate immune response	*Mus musculus*	Kidney		
PFHxS	humoral immune response mediated by circulating immunoglobulin	PFHxS results in decreased humoral immune response mediated by circulating immunoglobulin	*Homo sapiens*	Serum	Study type: Prospective birth cohort (n = 464 children) Exposure: Serum PFAS (three major compounds) at ages 5 and 7 Endpoints: Antibody concentrations against diphtheria and tetanus Key findings: Higher PFAS associated with lower antibody levels; combined PFAS exposure at ages 5 and 7 showed stronger effect	26041029 [54]
PFHxS	IgG immunoglobulin complex, circulating	PFHxS results in decreased IgG immunoglobulin complex, circulating	*Homo sapiens*	Serum	Study type: Prospective birth cohort (n = 516 at age 13) Exposure: Serum PFAS at ages 7 and 13 Endpoints: Antibodies against diphtheria and tetanus Key findings: Higher PFAS associated with lower diphtheria antibody levels; effect strongest for PFDA at age 7 and PFOA at age 13; few effects on tetanus antibodies	27501995 [55]

PFAS—per- and polyfluoroalkyl substances; PFOA—perfluorooctanoic acid; PFOS—perfluorooctanesulfonic acid; PFHxS—perfluorohexanesulfonic acid; IgG—immunoglobulin G.

**Table 3:** Molecular functions, biological processes, cellular components, molecular pathways, and diseases associated with genes common to all analyzed PFASs (ToppGene Suite; https://toppgene.cchmc.org, accessed on 12 June 2026).

	ID	Name	*p*-Value	Interacting Genes
Molecular functions	GO:0005102	Signal receptor binding	3.626 × 10^−10^	*TYK2*, *IFNG*, *IL10*, *TGFB2*, *S100A8*, *CD3E*, *IL18*, *FN1*, *CCN1*, *CD36*, *MBL2*, *SERPINE1*, *CASP8*, *TNF*, *APOE*, *IL1B*, *CBLB*, *GFAP*, *IL6*, *PCNA*, *SMAD3*, *IL27*, *MSN*.
	GO:0005126	Cytokine receptor binding	7.108 × 10^−9^	*TYK2*, *IFNG*, *IL10*, *TGFB2*, *IL18*, *CASP8*, *TNF*, *IL1B*, *IL6*, *SMAD3*, *IL27*.
	GO:0016209	Antioxidant activity	7.366 × 10^−7^	*S100A8*, *NQO1*, *CAT*, *ALB*, *APOE*, *SOD1*.
	GO:0005125	Cytokine activity	1.472 × 10^−6^	*IFNG*, *IL10*, *TGFB2*, *IL18*, *TNF*, *IL1B*, *IL6*, *IL27*.
	GO:0140677	Molecular function activator activity	1.477 × 10^−6^	*IFNG*, *IL10*, *TGFB2*, *ARHGAP4*, *IL18*, *FN1*, *CASP8*, *CCND1*, *TNF*, *APOE*, *IL1B*, *DDIT3*, *IL6*, *PCNA*, *IL27*, *FBXW7*.
	GO:0019902	Phosphatase binding	2.361 × 10^−5^	*KCNN4*, *BCL2*, *HMGCR*, *PPARA*, *PPARG*, *FAS*, *SOD1*, *SMAD3*.
	GO:0002020	Protease binding	2.706 × 10^−5^	*FN1*, *MBL2*, *SERPINE1*, *BCL2*, *TNF*, *FAS*.
	GO:0048018	Ligand receptor activity	6.639 × 10^−5^	*IFNG*, *IL10*, *TGFB2*, *IL18*, *FN1*, *TNF*, *APOE*, *IL1B*, *IL6*, *IL27*
	GO:0030546	Signal receptor activator activity	7.318 × 10^−5^	*IFNG*, *IL10*, *TGFB2*, *IL18*, *FN1*, *TNF*, *APOE*, *IL1B*, *IL6*, *IL27*.
	GO:0005504	Fatty acid binding	7.541 × 10^−5^	*S100A8*, *CD36*, *ALB*, *PPARG*.
Biological Processes	GO:0002682	Immune system process regulation	1.065 × 10^−27^	*TYK2*, *EPHB4*, *IFNG*, *IL10*, *TGFB2*, *S100A8*, *RAG1*, *RAG2*, *CD3E*, *IL18*, *PDE4B*, *FN1*, *CD14*, *NFE2L2*, *CD36*, *MBL2*, *SERPINE1*, *KCNN4*, *CASP8*, *SLC7A5*, *CARD11*, *BCL2*, *TNF*, *HMOX1*, *APOE*, *PPARG*, *IL1B*, *KAT6B*, *FAS*, *ADA*, *CBLB*, *RBM47*, *NOS2*, *ZAP70*, *IL6*, *VAV1*, *SOD1*, *SMAD3*, *IL27*, *FBXW7*, *MSN*.
	GO:0042981	Regulation of apoptosis	3.542 × 10^−25^	*IFNG*, *IL10*, *TGFB2*, *TXNIP*, *S100A8*, *RAG1*, *CD3E*, *IL18*, *FN1*, *CCN1*, *NFE2L2*, *CIDEC*, *MAZ*, *BAG3*, *SERPINE1*, *NQO1*, *CASP8*, *SLC7A5*, *CAT*, *CARD11*, *CCND1*, *BCL2*, *HMGCR*, *TNF*, *MMP2*, *PPARA*, *HMOX1*, *APOE*, *PPARG*, *IL1B*, *FAS*, *ADA*, *CBLB*, *NOS2*, *DDIT3*, *IL6*, *SOD1*, *SMAD3*, *FBXW7*.
	GO:0043067	Regulation of programmed cell death	1.032 × 10^−24^	*IFNG*, *IL10*, *TGFB2*, *TXNIP*, *S100A8*, *RAG1*, *CD3E*, *IL18*, *FN1*, *CCN1*, *NFE2L2*, *CIDEC*, *MAZ*, *BAG3*, *SERPINE1*, *NQO1*, *CASP8*, *SLC7A5*, *CAT*, *CARD11*, *CCND1*, *BCL2*, *HMGCR*, *TNF*, *MMP2*, *PPARA*, *HMOX1*, *APOE*, *PPARG*, *IL1B*, *FAS*, *ADA*, *CBLB*, *NOS2*, *DDIT3*, *IL6*, *SOD1*, *SMAD3*, *FBXW7*.
	GO:2000026	Regulation of multicellular organism development	9.688 × 10^−21^	*IFNG*, *IL10*, *TGFB2*, *RAG1*, *ARHGAP4*, *RAG2*, *IL18*, *FN1*, *CCN1*, *NFE2L2*, *GLI1*, *CD36*, *SERPINE1*, *CASP8*, *SLC7A5*, *CARD11*, *CCND1*, *TNF*, *PPARA*, *HMOX1*, *APOE*, *PPARG*, *IL1B*, *KAT6B*, *FAS*, *ADA*, *GFAP*, *ZAP70*, *IL6*, *B4GALT5*, *SOD1*, *SMAD3*, *IL27*, *FBXW7*, *STAB1*.
	GO:0001816	Cytokine production	1.549 × 10^−20^	*TYK2*, *IFNG*, *IL10*, *TGFB2*, *CD3E*, *IL18*, *PDE4B*, *FN1*, *CD14*, *CCN1*, *CD36*, *SERPINE1*, *NQO1*, *KCNN4*, *CASP8*, *SLC7A5*, *CARD11*, *TNF*, *PPARA*, *HMOX1*, *PPARG*, *IL1B*, *RBM47*, *NOS2*, *DDIT3*, *IL6*, *SOD1*, *SMAD3*, *IL27*.
	GO:0001817	Cytokine production regulation	2.975 × 10^−20^	*TYK2*, *IFNG*, *IL10*, *TGFB2*, *CD3E*, *IL18*, *PDE4B*, *FN1*, *CD14*, *CCN1*, *CD36*, *SERPINE1*, *KCNN4*, *CASP8*, *SLC7A5*, *CARD11*, *TNF*, *PPARA*, *HMOX1*, *PPARG*, *IL1B*, *RBM47*, *NOS2*, *DDIT3*, *IL6*, *SOD1*, *SMAD3*, *IL27*.
	GO:0001819	Positive regulation of cytokine production	8.699 × 10^−20^	*TYK2*, *IFNG*, *IL10*, *CD3E*, *IL18*, *PDE4B*, *CD14*, *CCN1*, *CD36*, *SERPINE1*, *CASP8*, *SLC7A5*, *CARD11*, *TNF*, *HMOX1*, *IL1B*, *RBM47*, *NOS2*, *DDIT3*, *IL6*, *SOD1*, *SMAD3*, *IL27*.
	GO:0050776	Regulation of immune response	1.987 × 10^−19^	*TYK2*, *IFNG*, *IL10*, *TGFB2*, *S100A8*, *CD3E*, *IL18*, *PDE4B*, *CD14*, *NFE2L2*, *CD36*, *MBL2*, *KCNN4*, *CASP8*, *CARD11*, *BCL2*, *TNF*, *APOE*, *PPARG*, *IL1B*, *ADA*, *CBLB*, *RBM47*, *NOS2*, *ZAP70*, *IL6*, *VAV1*, *SMAD3*, *IL27*.
	GO:0043069	Negative regulation of programmed cell death	1.482 × 10^−18^	*IL10*, *TGFB2*, *RAG1*, *IL18*, *FN1*, *CCN1*, *NFE2L2*, *MAZ*, *BAG3*, *SERPINE1*, *NQO1*, *CASP8*, *SLC7A5*, *CAT*, *CCND1*, *BCL2*, *HMGCR*, *TNF*, *PPARA*, *HMOX1*, *APOE*, *IL1B*, *FAS*, *ADA*, *CBLB*, *IL6*, *SOD1*, *SMAD3*.
	GO:0050865	Regulation of cell activation	1.515 × 10^−18^	*TYK2*, *EPHB4*, *IFNG*, *IL10*, *RAG1*, *RAG2*, *CD3E*, *IL18*, *FN1*, *KCNN4*, *CARD11*, *BCL2*, *TNF*, *APOE*, *PPARG*, *IL1B*, *FAS*, *ADA*, *CBLB*, *ZAP70*, *IL6*, *VAV1*, *SOD1*, *IL27*.
Cellular components	GO:0098552	Membrane surface	7.324 × 10^−9^	*TYK2*, *IFNG*, *CD3E*, *CD14*, *CD36*, *MBL2*, *SLC7A5*, *TNF*, *ABCG2*, *APOE*, *FAS*, *ADA*, *GFAP*, *IL6*, *MSN*.
	GO:0009897	External side of plasma membrane	5.888 × 10^−8^	*IFNG*, *CD3E*, *CD14*, *CD36*, *MBL2*, *SLC7A5*, *TNF*, *ABCG2*, *APOE*, *FAS*, *ADA*, *IL6*.
	GO:0009986	Cell surface	1.174 × 10^−6^	*IFNG*, *TGFB2*, *CD3E*, *CD14*, *CD36*, *MBL2*, *SLC7A5*, *TNF*, *ABCG2*, *APOE*, *FAS*, *ADA*, *IL6*, *IL27*, *MSN*
	GO:0045121	Membrane raft	1.725 × 10^−6^	*CD14*, *CD36*, *CARD11*, *TNF*, *ABCG2*, *HMOX1*, *FAS*, *CBLB*, *ZAP70*.
	GO:0098857	Membrane microdomain	1.805 × 10^−6^	*CD14*, *CD36*, *CARD11*, *TNF*, *ABCG2*, *HMOX1*, *FAS*, *CBLB*, *ZAP70*.
	GO:0097519	DNA recombinase complex	9.520 × 10^−6^	*RAG1*, *RAG2*.
	GO:0031091	Platelet alpha-granules	1.046 × 10^−5^	*TGFB2*, *FN1*, *CD36*, *SERPINE1*, *ALB*.
	GO:0044297	Cell soma	2.528 × 10^−5^	*IFNG*, *TGFB2*, *CD3E*, *NQO1*, *KCNN4*, *CASP8*, *TNF*, *APOE*, *FAS*, *ADA*, *GFAP*, *SOD1*.
	GO:0031093	Luminal alpha-granules of platelets	5.632 × 10^−5^	*TGFB2*, *FN1*, *SERPINE1*, *ALB*.
	GO:0030141	Secretory granules	5.926 × 10^−5^	*TGFB2*, *S100A8*, *FN1*, *CD14*, *CD36*, *SERPINE1*, *CAT*, *TNF*, *ALB*, *IL1B*, *FAS*, *SOD1*.
Pathways	M874	Interleukin signaling pathway	2.252 × 10^−17^	*TYK2*, *IFNG*, *IL10*, *RAG1*, *RAG2*, *IL18*, *FN1*, *CD36*, *CASP8*, *CCND1*, *BCL2*, *TNF*, *MMP2*, *HMOX1*, *IL1B*, *NOS2*, *IL6*, *VAV1*, *SOD1*, *SMAD3*, *IL27*, *MSN*.
	M27609	Interleukin 4 and 13 signaling pathway	1.309 × 10^−15^	*TYK2*, *IL10*, *TGFB2*, *IL18*, *FN1*, *CD36*, *CCND1*, *BCL2*, *TNF*, *MMP2*, *HMOX1*, *IL1B*, *NOS2*, *IL6*.
	M48052	Urotensin-mediated signaling pathway	3.521 × 10^−13^	*FN1*, *BCL2*, *TNF*, *MMP2*, *HMOX1*, *IL1B*, *DDIT3*, *IL6*, *PCNA*, *SMAD3*.
	M42569	WP SARS-CoV-2 signaling network map	4.447 × 10^−13^	*IFNG*, *IL10*, *CD3E*, *IL18*, *FN1*, *CD14*, *SERPINE1*, *CASP8*, *CARD11*, *TNF*, *ALB*, *IL1B*, *ZAP70*, *IL6*.
	M39818	WP IL18 signaling pathway	5.673 × 10^−13^	*IFNG*, *IL10*, *IL18*, *FN1*, *CD36*, *CASP8*, *BCL2*, *TNF*, *MMP2*, *HMOX1*, *IL1B*, *FAS*, *NOS2*, *IL6*, *FBXW7*.
	M1060	Cytokine signaling pathway in the immune system	8.710 × 10^−13^	*TYK2*, *IFNG*, *IL10*, *RAG1*, *RAG2*, *IL18*, *FN1*, *CD36*, *CASP8*, *CCND1*, *BCL2*, *TNF*, *MMP2*, *HMOX1*, *IL1B*, *NOS2*, *IL6*, *VAV1*, *SOD1*, *SMAD3*, *IL27*, *MSN*.
	M196	PID IL23 pathway	3.063 × 10^−12^	*TYK2*, *IFNG*, *CD3E*, *IL18*, *TNF*, *IL1B*, *NOS2*, *IL6*.
	M48313	WP post-COVID neuroinflammation	5.208 × 10^−12^	*IFNG*, *IL10*, *TGFB2*, *IL18*, *FN1*, *CD36*, *CASP8*, *BCL2*, *TNF*, *MMP2*, *HMOX1*, *IL1B*, *FAS*, *NOS2*, *IL6*, *GFAP*.
	M36	PID IL27 pathway	1.383 × 10^−11^	*TYK2*, *IFNG*, *IL18*, *TNF*, *IL1B*, *IL6*, *IL27*.
	M39438	WP survival signaling pathway mediated by AP1	4.816 × 10^−11^	*IFNG*, *NFE2L2*, *CCND1*, *BCL2*, *TNF*, *MMP2*, *FAS*, *IL6*.
Diseases	DOID:13241	Behçet’s disease	4.424 × 10^−27^	*IFNG*, *IL10*, *IL18*, *MBL2*, *SERPINE1*, *CAT*, *TNF*, *MMP2*, *HMOX1*, *IL1B*, *FAS*, *IL6*, *SOD1*.
	C0011853	Experimental diabetes mellitus	1.836 × 10^−23^	*IFNG*, *SERPINE1*, *NQO1*, *CAT*, *BCL2*, *TNF*, *MMP2*, *PPARA*, *HMOX1*, *PPARG*, *IL1B*, *FAS*, *NOS2*, *IL6*, *SOD1*.
	C0002152	Alloxan diabetes	1.836 × 10^−23^	*IFNG*, *SERPINE1*, *NQO1*, *CAT*, *BCL2*, *TNF*, *MMP2*, *PPARA*, *HMOX1*, *PPARG*, *IL1B*, *FAS*, *NOS2*, *IL6*, *SOD1*.
	C0038433	Streptozotocin diabetes	1.836 × 10^−23^	*IFNG*, *SERPINE1*, *NQO1*, *CAT*, *BCL2*, *TNF*, *MMP2*, *PPARA*, *HMOX1*, *PPARG*, *IL1B*, *FAS*, *NOS2*, *IL6*, *SOD1*.
	C0020538	Hypertensive disease	2.260 × 10^−21^	*FN1*, *CD36*, *SERPINE1*, *CAT*, *BCL2*, *TNF*, *ALB*, *MMP2*, *PPARA*, *HMOX1*, *APOE*, *PPARG*, *IL1B*, *NOS2*, *IL6*, *SOD1*.
	C0035126	Reperfusion injury	9.901 × 10^−21^	*IL10*, *CD36*, *CASP8*, *CAT*, *BCL2*, *TNF*, *PPARA*, *HMOX1*, *PPARG*, *IL1B*, *NOS2*, *IL6*, *SOD1*.
	DOID-2841	Asthma	1.131 × 10^−18^	*IL10*, *TGFB2*, *IL18*, *CD14*, *NFE2L2*, *SERPINE1*, *CAT*, *TNF*, *HMOX1*, *IL1B*, *NOS2*, *IL6*, *IL27*.
	C0003873	Rheumatoid arthritis	1.697 × 10^−18^	*TYK2*, *IFNG*, *IL10*, *TXNIP*, *CD3E*, *IL18*, *CCN1*, *TFPI2*, *CAT*, *TNF*, *ABCG2*, *MMP2*, *IL1B*, *IL6*.
	DOID:10763	Hypertension	1.810 × 10^−18^	*IL10*, *FN1*, *NFE2L2*, *CD36*, *SERPINE1*, *CASP8*, *CAT*, *BCL2*, *MMP2*, *PPARA*, *APOE*, *PPARG*, *IL1B*, *NOS2*, *SOD1*.
	DOID:12361	Graves’ disease	2.254 × 10^−18^	*IFNG*, *IL10*, *IL18*, *SERPINE1*, *CAT*, *IL1B*, *FAS*, *IL6*.

GO—Gene Ontology; WP—WikiPathways; PID—Pathway Interaction Database; DOID—Disease Ontology Identifier; IL—interleukin; AP1—activator protein 1.

**Table 4:** Functional enrichment analysis of MCODE components identified within the protein–protein interaction network of genes common to all analyzed PFAS compounds.

MCODE Cluster	GO/Pathway ID	Description	−log10(P)
MCODE_1	WP1533	Vitamin B12 metabolism	6.5
MCODE_1	GO:2001244	Positive regulation of intrinsic apoptotic signaling pathway	6.2
MCODE_1	WP176	Folate metabolism	6.2
MCODE_2	WP5485	Post-COVID neuroinflammation	18.1
MCODE_2	WP5561	Integrative Analysis of Treg Glial Interactions	17.1
MCODE_2	M196	PID IL23 pathway	16.8
MCODE_3	hsa05162	Measles	7.1
MCODE_3	WP4630	Measles virus infection	7.1
MCODE_4	GO:0043535	Regulation of blood vessel endothelial cell migration	6.9
MCODE_4	GO:0010594	Regulation of endothelial cell migration	6.3
MCODE_4	GO:0045765	Regulation of angiogenesis	5.7
NA	R-HSA-449147	Signaling by Interleukins	−24.6
NA	R-HSA-1280215	Cytokine Signaling in Immune system	−19.7
NA	R-HSA-6785807	Interleukin-4 and Interleukin-13 signaling	−18.6

NA—not available.

## Data Availability

The original contributions presented in this study are included in the article. Further inquiries can be directed to the corresponding author.

## Abbreviations

The following abbreviations are used in this manuscript:

PFAS	Per- and polyfluoroalkyl substances
PFOA	Perfluorooctanoic acid
PFNA	Perfluorononanoic acid
PFHxS	Perfluorohexanesulfonic acid
PFOS	Perfluorooctanesulfonic acid
CTD	Comparative Toxicogenomic Database
OECD	Organisation for Economic Co-operation and Development
EFSA	European Food Safety Authority
FDR	False discovery rate
MCODE	Molecular Complex Detection
MCC	Maximum Clique Centrality
ME	mRNA expression
PE	Protein expression
NA	Not available
